# Comparing the Effects of Pre-loading with Gelatine 4% Plasma Volume Expander and 6% Hydroxyethyl Starch Solution Before Spinal Anaesthesia for Lower Limb Orthopaedic Surgery

**DOI:** 10.21315/mjms2020.27.6.7

**Published:** 2020-12-29

**Authors:** Nur Dyana Md Nizar, Shamsul Kamalrujan Hassan, Rhendra Hardy Mohamad Zaini, Mohamad Hasyizan Hassan, Wan Mohd Nazaruddin Wan Hassan, Mohd Zulfakar Mazlan

**Affiliations:** 1 Department of Anaesthesiology & Intensive Care, School of Medical Sciences, Universiti Sains Malaysia, Kelantan, Malaysia; 2 Department of Anaesthesiology & Intensive Care, Hospital Universiti Sains Malaysia, Kelantan, Malaysia

**Keywords:** post-spinal hypotension, starch, colloids, vital signs, acid-base, electrolytes

## Abstract

**Background:**

Hypotension is a common complication following spinal anaesthesia. The administration of intravenous fluids prior to spinal anaesthesia, known as pre-loading, has been used to offset the hypotension effect; however, the ideal fluid for pre-loading is still a matter of debate. The objective of this study was to compare the effects of Gelaspan 4% and Volulyte 6% as pre-loading fluids.

**Methods:**

A total of 93 patients with American Society of Anaesthesiologists (ASA) physical status I or II having lower limb orthopaedic surgery under spinal anaesthesia were randomised into two groups that received either Volulyte (*n =* 47) or Gelaspan (*n =* 46). Before the spinal anaesthesia, these patients were pre-loaded with 500 mL of the fluid of their respective group. Blood samples were taken before pre-loading and again after spinal anaesthesia and sent for venous blood gas and electrolyte level measurement. Baseline and intraoperative records of systolic blood pressure (SBP), diastolic blood pressure (DBP), mean arterial pressure (MAP), heart rate (HR) and the requirement of ephedrine to treat hypotension were also recorded.

**Results:**

Both fluids could not prevent significant reductions in SBP (*P =* 0.011), DBP (*P =* 0.002) and MAP (*P =* 0.001). There was also significant reduction in HR over time (*P <* 0.001). There was no significant difference in terms of ephedrine usage between both groups. Neither Volulyte 6% nor Gelaspan 4% caused significant changes in acid-base status.

**Conclusion:**

The use of 500 mL of either Gelaspan 4% or Volulyte 6% as pre-loading fluids did not significantly prevent the incidence of post-spinal anaesthesia hypotension following orthopaedic lower limb surgery; however, both were useful in the maintenance normal acid-base balance.

## Introduction

Spinal anaesthesia is the method of choice for the anaesthesia in lower limb orthopaedic surgery. Despite its many benefits, however, spinal anaesthesia has common complications, such as hypotension and bradycardia (1). These complications mainly occur due to the sympathetic blockade leading to peripheral vasodilation and the venous pooling of blood. As a result, there is a decrease in venous return and cardiac output that leads to hypotension (2). The spectrum of morbidities associated with hypotension include nausea, vomiting, dizziness, syncope, cardiac arrhythmia and even cardiac arrest (3).

One of the foremost methods for preventing hypotension following spinal anaesthesia includes the administration of intravenous fluids before the implementation of the subarachnoid block (4, 5). Pre-loading with crystalloids up to 30 mL/kg only moderately prevents hypotension (6), while colloid pre-loading is said to be more reliable as it sustains intravascularly longer (7–8). However, the ideal fluid for pre-loading is still a matter of debate. Volulyte contains 6% hydroxyethyl starch (HES 130/0.4), which is a tetrastarch that is a derivative of waxy maize starch. The infusion of 500 mL HES 130/0.4 over 30 min in healthy volunteers results in plasma expansion for approximately 4–6 h. Volulyte contains the electrolytes sodium, potassium, magnesium, chloride and acetate in an isotonic composition. On the other hand, Gelaspan contains 40 g succinylated gelatine, which is characterised by long stretched polypeptide chains and an increased negative charge created by succinylation. Having an in vivo strong ion difference derived from both the metabolism of organic anions and gelatine’s charge, this solution may have a less acidifying effect than the older generation solution known as Gelofusine.

Both Gelaspan and Volulyte contain acetate, which metabolises faster than other colloids. For every mole of acetate or lactate oxidised, one mole of bicarbonate is produced, which is then metabolised in the liver and muscles and the potential base excess of the solution is 0 mmol/L. Thus, after the infusion and metabolism of acetate, these solutions do not affect the patient’s acid-base balance. In contrast to Volulyte, Gelaspan contains a higher concentration of sodium and a lower concentration of acetate. Furthermore, Gelaspan contains calcium, which is not found in Volulyte.

In the crystalloid versus HES trial (CHEST) study, the use of colloids was found to be associated with a higher risk of acute kidney injury in burn and sepsis patients, leading to a refraining from its use in the clinical practice of intensive care units (9). Nevertheless, the benefits of colloids in the prevention and management of intraoperative spinal anaesthesia-induced hypotension are clear (8). The use of colloids as pre-loading fluids in the elective lower abdominal, lower limb and gynaecological procedures is effective in preventing post-spinal anaesthesia induced hypotension (10). There are different kinds of colloids available for clinical practice; however, there have been no studies comparing these different colloids. Therefore, this study aimed to compare two colloids and their benefits in preventing the hazard of post-spinal anaesthesia hypotension.

## Methods

A total of 93 patients aged 18–60 years old with an American Society of Anesthesiologists (ASA) physical status of either I or II who underwent elective or emergency orthopaedic lower limb surgery under spinal anaesthesia at Hospital Universiti Sains Malaysia between November 2017 and October 2018 were selected for this study. The patients were randomised into two groups, one receiving Volulyte (*n* = 47) and the other Gelaspan (*n* = 46). Patients were excluded from the study if they had a BMI > 35, height less than 140 cm, history of neurological or psychiatric diseases, contraindication to spinal anaesthesia, known allergy to Gelaspan or Volulyte or septic shock. Patients were withdrawn from the study if they had failed spinal anaesthesia, if the spinal anaesthesia was complicated with total spinal anaesthesia or if the patient developed an allergic reaction to Gelaspan and Volulyte.

The solutions were covered with black paper and labelled A or B to ensure blinding and remained covered throughout the surgery. Therefore, the investigator and the patients remained blind to which solution was used. Demographic data, baseline and intraoperative records of systolic blood pressure (SBP), diastolic blood pressure (DBP), mean arterial pressure (MAP) and heart rate (HR), the requirement of ephedrine to treat hypotension and the pH, bicarbonate, base excess, sodium, calcium and potassium levels from venous blood gas were documented in the observational checklist. Patients were connected to standard devices for electrocardiography, pulse oximetry and non-invasive blood pressure monitoring. Baseline variables were measured and charted. One 18G branula was inserted for drug administration, rapid pre-loading and maintenance. A 1 mL blood sample for VBG, lactate and electrolytes was taken during the insertion of the branula. Both groups received a pre-load of 500 mL of either Gelaspan or Volulyte solution within the 30 min immediately before receiving the spinal anaesthesia.

The patient was placed in a sitting position for the spinal anaesthesia after completing the pre-loading regime. The subarachnoid puncture was done with a 25-gauge Spinocan needle at the L3–L4 space by using the midline approach, and 2.6 mL of 0.5% heavy bupivacaine and 20 mcg of fentanyl were injected into the subarachnoid space. The patient was then put in a supine position. Sensory dermatome levels and motor power were tested to determine the distribution of the effect of the local anaesthesia. The aim in all patients was to achieve sensory and motor blockade up to the navel area or equal to thoracic 10 distribution.

Oxygen at 5 L/min was administered via a facemask. Blood pressure was then measured with the automated blood pressure device immediately post the spinal anaesthesia and then every 5 min for 30 min. Hypotension is defined as a MAP of less than 65 mmHg or a drop in blood pressure > 20% from baseline. A 3 mg bolus of ephedrine was given in the case of hypotension, which was repeated every 2 min if hypotension persisted or recurred. The incidence of hypotension and the amount of ephedrine required to treat hypotension were compared in both groups. An IV drip of normal saline was used for fluid maintenance intraoperatively. Another 1 mL blood sample (VBG) was taken immediately post the subarachnoid block.

The sample size was calculated using G*Power software (Version 3.1.9.2, Universitat Kiel, Germany). Based on the objective of comparing the haemodynamic changes in both groups (SBP, DBP, MAP and HR) and using an effect size of 0.4, an α error probability, a power of 0.8, two groups and seven measurements, 44 subjects per arm were needed to reject the null hypothesis. Thus, including a possible 10% drop out, the total sample size was figured as 96 patients.

The data were analysed using IBM SPSS Statistics software version 24. All measurement data were analysed for normal distribution and homogeneity variance. The demographic data were analysed using an independent *t*-test for the numerical data and Chi-square analysis for the categorical data. A two-way repeated measures ANOVA (mixed-design) was conducted to determine whether there were significant differences between the Volulyte and Gelaspan groups for SBP, DBP, MAP and HR at seven time-points (baseline and 5 min, 10 min, 15 min, 20 min, 25 min and 30 min post-spinal anaesthesia). Model assumptions of normality, homogeneity of covariance and compound symmetry were checked. Besides, electrolytes and acid-base status were compared within each group using paired *t*-tests and comparisons between the groups using independent *t*-tests. A *P*-value < 0.05 was considered as a significant difference.

## Results

A total of 96 patients were enrolled in this study. Of these, three patients were excluded due to failed spinal anaesthesia and so 93 patients were included in the final analysis (47 Volulyte, 46 Gelaspan). There were no significant differences in terms of age, gender, weight, height or BMI between the Volulyte 6% and Gelaspan 4% groups (Table 1). There were also no significant differences between the baseline haemodynamic statuses of the two groups (Table 2).

Based on the two-way repeated-measures ANOVA analysis, there was no significant difference overall in mean SBP between Volulyte and Gelaspan in regards of time [*F* (1, 91 = 0.59; *P* = 0.444)]. There was, however, a significant interaction between each fluid and time for SBP [*F* (2.954, 268.779 = 3.381; *P* = 0.011)]. Therefore, stratification according to the groups was done during the data analysis (Table 3).

For Volulyte, Mauchly’s test for sphericity indicated that the assumption was not met (Chi-square = 153.792, df = 20, *P* < 0.001), so a Greenhouse-Geisser correction was applied. There was an overall significant change in SBP over time [*F* (2.564, 117.95 = 2.880; *P* = 0.047)], where a post-hoc paired *t*-test with a Bonferroni correction showed a significant reduction in SBP at 5 min and 30 min (mean difference = 6.7, 95% CI: 2.04, 22.37, *P* = 0.001) post-spinal anaesthesia.

For Gelaspan, Mauchly’s test for sphericity also indicated that the assumption was not met (Chi-square = 101.574, df = 20, *P* < 0.001), so a Greenhouse-Geisser correction was applied. There was a significant change in overall SBP over time [*F* (3.207, 144.321 = 7.013; *P* < 0.001)]; however, a post-hoc paired *t*-test with a Bonferroni correction showed no significant reduction in SBP for any comparison.

The results of the two-way repeated-measures ANOVA also revealed no significant difference overall in mean DBP between Volulyte and Gelaspan in regards of time [*F* (1, 91 = 1.662; *P* = 0.201)]. However, there was a significant interaction between each fluid and time for DBP [*F* (2.953, 268.694 = 5.228; *P* = 0.002)]. Thus, stratification according to the group was again performed during the data analysis (Table 4).

For Volulyte, Mauchly’s test for sphericity indicated that the assumption was not met (Chi-square = 151.106, df = 20, *P* < 0.001), so a Greenhouse-Geisser correction was applied. There was a significant change in overall DBP over time [*F* (2.567, 118.102 = 3.749; *P* = 0.018)]; however, a post-hoc paired *t*-test with a Bonferroni correction showed no significant change in mean DBP for any comparison.

For Gelaspan, Mauchly’s test for sphericity also indicated that the assumption was not met (Chi-square = 114.466, df = 20, *P* < 0.001), so a Greenhouse-Geisser correction was again applied. There was a significant change in overall DBP over time [*F* (3.209, 144.391 = 15.288; *P* < 0.001)], where a post-hoc paired *t-*test with a Bonferroni correction showed significant reductions in DBP between baseline and 10 min (mean difference = 8.4, 95% CI: 2.58, 14.29), 15 min (mean difference = 8.8, 95% CI: 2.69, 14.84), 20 min (mean difference = 10.1, 95% CI: 4.70, 15.52), 25 min (mean difference = 9.3, 95% CI: 3.69, 14.96) and 30 min (mean difference = 9.7, 95% CI: 3.78, 15.66) post-spinal anaesthesia.

The two-way repeated-measures ANOVA also showed no significant difference overall in mean MAP between Volulyte and Gelaspan in regards of time [*F* (1, 91 = 1.734; *P* = 0.191)]. However, there was a significant interaction between each fluid and time for MAP [*F* (2.709, 246.519 = 5.865; *P* = 0.001)]. Therefore, stratification according to the group was again done during the data analysis (Table 5).

For Volulyte, Mauchly’s test for sphericity indicated that the assumption was not met (Chi-square = 187.500, df = 20, *P* < 0.001), so a Greenhouse-Geisser correction was applied. There was an significant change in overall MAP over time [*F* (2.153, 99.039 = 4.342; *P* = 0.014)], where a post-hoc paired *t*-test with a Bonferroni correction showed significant changes in mean MAP between 5 min and 30 min (mean difference = 6.2, 95% CI: 2.35, 10.08) and 10 min and 30 min (mean difference = 4.6, 95% CI: 1.20, 7.95) post-spinal anaesthesia.

For Gelaspan, Mauchly’s test for sphericity also indicated that the assumption was not met (Chi-square = 117.422, df = 20, *P* < 0.001), so a Greenhouse-Geisser correction was applied. There was a significant change in overall MAP over time [*F* (3.220, 144.898 = 6.911; *P* > 0.001)]; however, a post-hoc paired *t*-test with a Bonferroni correction showed no significant change in mean MAP for any comparison.

For HR, Mauchly’s test for sphericity indicated that the assumption was not met (Chi-square = 301.571, df = 20, *P* < 0.001), so a Greenhouse-Geisser correction was applied. There was an significant change in overall HR over time [*F* (2.837, 258.186 = 19.613; *P* < 0.001)], where a post-hoc paired *t*-test with a Bonferroni correction showed significant changes in mean HR between baseline and 20 min (mean difference = 5.0; 95% CI: 0.96, 9.08), baseline and 25 min (mean difference = 6.6; 95% CI: 2.62, 10.60), 5 min and 15 min (mean difference = 3.5; 95% CI: 1.57, 5.47), 5 min and 20 min (mean difference = 4.9; 95% CI: 2.20, 7.64), 5 min and 25 min (mean difference = 6.5; 95% CI: 3.83, 9.19), 5 min and 30 min (mean difference = 6.3; 95% CI: 3.12, 9.39), 10 min and 15 min (mean difference = 2.3; 95% CI: 0.76, 3.83), 10 min and 20 min (mean difference = 3.7; 95% CI: 1.39, 6.00), 10 min and 25 min (mean difference = 5.3; 95% CI: 2.88, 7.69), 10 min and 30 min (mean difference = 5.0; 95% CI: 2.25, 7.82), 15 min and 25 min (mean difference = 3.0; 95% CI: 1.19, 4.79), 15 min and 30 min (mean difference = 2.7; 95% CI: 0.52, 4.96) and 20 min and 25 min (mean difference = 1.6; 95% CI: 0.07, 3.11) post-spinal anaesthesia. There was no overall significant difference in mean HR between Volulyte and Gelaspan in regards of time [*F* (1, 91 = 0.047; *P* = 0.829)]. Furthermore, there was no significant interaction between either fluid and time for HR [*F* (2.837, 258.186 = 1.560; *P* = 0.202)] (Table 6).

In this study, only one case required ephedrine for the treatment of hypotension, which was in the Gelaspan 4% group.

There were no baseline differences in terms of acid-base status and electrolytes between the two groups. Overall, fluid pre-loading with either Volulyte 6% or Gelaspan 4% did not cause changes in the acid-base status of the patients. Sodium decreased somewhat more in the Volulyte 6% group than in the Gelaspan 4% group (*P* = 0.037). Calcium concentration was significantly lower after pre-loading with Volulyte 6% than with Gelaspan 4% (*P* = 0.018).

There was no significant difference in acid-base balance and electrolytes after pre-loading with Volulyte 6% (Table 7).

There were significant increments in the mean baseline and post-intervention calcium and potassium levels in the Gelaspan group. Mean calcium and potassium were significantly higher after pre-loading with Gelaspan than at baseline, with *P*-values of 0.017 and 0.015, respectively (Table 8). There was also a significant reduction in pH after pre-loading with Gelaspan 4% (*P* = 0.029); however, it remained within normal limits.

## Discussion

Hypotension is a common complication following spinal anaesthesia and occurs because the sympathetic blockade leads to vasodilation, relative hypovolaemia and decreased venous return. Cementation of the prosthesis and deflation of the tourniquet are said to be among the risk factors (1). Colloids are believed to be more effective in maintaining arterial blood pressure than crystalloids because they augment intravascular volume better and longer than crystalloids, and they are thus more effective in preventing hypotension in spinal anaesthesia (8, 10, 11)

Using arterial pulse contour analysis, a recent study compared the effects of pre-loading 0.5 L 6% HES 130/0.42 versus 1 L Ringer’s lactate on the haemodynamic status of parturient patients undergoing spinal anaesthesia for elective caesarean delivery and showed that pre-loading with 0.5 L HES 130/0.42 produced a more stable haemodynamic situation than 1 L Ringer’s lactate solution in these patients (12).

In our study, we limited our pre-loading to 500 mL of colloid. Afterwards, we continued fluid maintenance with crystalloid. There was still a significant reduction in blood pressure despite pre-loading with 500 mL colloid, whether in the Gelaspan 4% group or Volulyte 6% group, especially at the end of the 30 min study. However, the means for SBP, DBP and MAP all remained within the normal range. For Volulyte, the difference from the baseline of MAP at 5 min and 30 min post-spinal anaesthesia was 6.7 mmHg. In clinical practice, the difference between a MAP of 91 mmHg and 97 mmHg is not significant; however, a 6.7 mmHg change is significant if it is a comparison between a MAP of less than 65 mmHg and more than 65 mmHg. An intervention such as ephedrine is required to keep MAP greater than 65 mmHg and prevent impaired tissue perfusion to vital organs, such as the heart, kidneys, brain and liver.

Besides, only one case in this study required ephedrine use. A recent study showed that pre-loading with approximately 700 mL of colloid is required to prevent post-spinal anaesthesia hypotension in 50% of the parturient (13). A randomised controlled trial by Tamilselvan et al. in 2019 (14) on the effects of crystalloid and colloid pre-load on cardiac output in the parturient undergoing planned caesarean delivery under spinal anaesthesia revealed that, while cardiac output and corrected flow time (a measure of intravascular volume) increased after pre-load in all groups, it was only maintained within HES 1 L group after spinal anaesthesia. Nevertheless, there were no differences among the groups in the incidence of hypotension or mean ephedrine use. This data suggests that, while cardiac output increases after the pre-load, it still cannot compensate for the reduction in blood pressure due to vasodilation following spinal anaesthesia.

Our intergroup analysis showed that there was a statistically significant difference in sodium levels post-preload. Post-preload sodium was higher in the Gelaspan group as compared to the Volulyte group (*P* = 0.037). This can be explained by the higher sodium content of Gelaspan, which contains 151 mmol/L, while Volulyte contains 137 mmol/L sodium. This finding is consistent with the previous study done by Krzych and Czempik in 2017 (15).

The post-preload calcium level was also significantly higher in the Gelaspan group (*P* = 0.018). The significantly higher value in the Gelaspan group as compared to the Volulyte group is probably because Gelaspan contains 1 mmol/L calcium, which is in contrast with Volulyte that contains no calcium. A study done by Tungria et al. (16) demonstrated no appreciable effect on serum sodium but did find a drop in serum calcium with the use of 6% HES 130 in gynaecological surgery patients. The use of a balanced colloid causes less acidosis (17–19). In our study, both Gelaspan and Volulyte did not cause significant acidosis. Thus, the use of a balanced colloid improves the acid-base status of the patient while also providing good haemodynamic stabilisation.

## Limitations

There were several limitations to our study. First, the study included only ASA I and ASA II patients with BMIs lower than 35 kg/m^2^ undergoing lower limb orthopaedic surgery. High-risk patients with ASA statuses III and above, obese patients with BMIs greater than 35 kg/m^2^ and heights less than 140 cm were excluded from this study as the effects of spinal anaesthesia in these groups of patients will be variable and risky. Therefore, the results of this study may not apply to the general population. Another potential limitation to this study is that we limited the pre-load volume to 500 mL of colloid. Ueyama et al. (6) reported that increasing the HES pre-load from 500 mL to 1000 mL dramatically improved its efficacy in preventing post-spinal anaesthesia hypotension. The third limitation of this study is that we did not study the effect of both colloids on renal function and coagulation profile.

Until now, the ideal fluids of choice for use as pre-loading fluids before spinal anaesthesia in lower limb surgery had not been studied much. Neither Gelaspan nor Volulyte offered better haemodynamic changes following spinal anaesthesia. The evidence for colloid use is limited, and both colloids and crystalloids have not revealed improved mortality or morbidity (20). For prompt resuscitation, however, colloids offer the ability to expand intravascular volume 1.5 times that of crystalloids (21–22).

## Conclusion

The use of colloids such as Gelaspan and Volulyte causes less significant post-spinal anaesthesia hypotension. Both colloids are useful as pre-loading fluids before spinal anaesthesia in orthopaedic surgery as well as for preventing acid-base balance disturbance and electrolyte derangement.

## Figures and Tables

**Table 1:** Patient demographics (*n* = 93)

Variable	Volulyte 6% (*n* = 47) *n* (%)	Gelaspan 4% (*n* = 46) *n* (%)	*P*-value
Age (years) *	39.2 (13.60)	37.5 (15.33)	0.585^a^
Gender			
Male	30 (48.4)	32 (51.6)	0.557^b^
Female	17 (54.8)	14 (45.2)	
Weight (kg)*	67.0 (14.00)	66.3 (9.57)	0.800^a^
Height (cm)*	164.9 (8.18)	162.4 (6.62)	0.103^a^
BMI (kg/m^2^) *	24.3 (4.58)	24.9 (4.09)	0.473^a^

Note: BMI = body mass index;

* mean (SD);

a Independent *t*-test;

b Chi-square test

**Table 2:** Comparison of baseline hemodynamic between Volulyte 6% and Gelaspan 4%

Variable	Mean (SD)		*P*-value^a^
	Volulyte 6% (*n* = 47)	Gelaspan 4% (*n* = 46)	
SBP (mmHg)	127.8 (14.94)	133.2 (17.80)	0.113
DBP (mmHg)	72.8 (10.62)	76.5 (10.22)	0.086
MAP (mmHg)	91.2 (10.92)	95.5 (10.84)	0.061
HR (bpm)	81.9 (14.75)	83.7 (11.75)	0.507

Notes: SBP = systolic blood pressure; DBP = diastolic blood pressure; MAP = mean arterial pressure; HR = heart rate; bpm = beats per minute;

a Independent *t*-test

**Table 3:** Comparison of SBP between Volulyte 6% and Gelaspan 4% in relation to time

Time	Mean (95% CI)^a^	
	Volulyte 6% (*n* = 47)	Gelaspan 4% (*n* = 46)
Baseline	127.8 (123.38, 132.15)	133.2 (127.93, 138.50)
5 min after pre-load	132.7 (126.87, 138.58)	126.4 (120.78, 132.05)
10 min after pre-load	128.9 (122.98, 134.90)	123.7 (117.86, 129.57)
15 min after pre-load	127.3 (120.96, 133.68)	123.0 (117.01, 128.95)
20 min after pre-load	126.4 (120.25, 132.51)	123.5 (117.57, 129.39)
25 min after pre-load	126.7 (120.45, 132.87)	123.3 (116.84, 129.73)
30 min after pre-load	126.0 (120.37, 131.67)	122.8 (116.40, 129.16)

Note:

a Two-way ANOVA

**Table 4:** Comparison of DBP between Volulyte 6% and Gelaspan 4% in relation to time

Time	Mean (95% CI)^a^	
	Volulyte 6% (*n* = 47)	Gelaspan 4% (*n* = 47)
Baseline	72.8 (69.65, 75.88)	76.5 (73.49, 79.56)
5 min after pre-load	73.9 (70.25, 77.54)	71.4 (68.26, 74.53)
10 min after pre-load	73.2 (69.61, 76.77)	68.1 (64.93, 71.25)
15 min after pre-load	71.5 (67.88, 75.01)	67.8 (64.94, 70.58)
20 min after pre-load	70.6 (67.29, 73.99)	66.4 (63.84, 68.98)
25 min after pre-load	69.9 (66.51, 73.24)	67.2 (64.21, 70.18)
30 min after pre-load	69.7 (66.28, 73.04)	66.8 (63.98, 69.63)

Note:

a Two-way ANOVA

**Table 5:** Comparison of MAP between Volulyte 6% and Gelaspan 4% group with regards to time

Time	Mean (95% CI)^a^	
	Volulyte 6% (*n* = 47)	Gelaspan 4% (*n* = 47)
Baseline	91.2 (87.96, 94.38)	95.5 (92.24, 98.68)
5 min after pre-load	97.2 (92.50, 101.84)	92.1 (88.27, 95.86)
10 min after pre-load	95.5 (90.85, 100.21)	89.6 (85.72, 93.50)
15 min after pre-load	94.4 (89.29, 99.61)	88.8 (85.12, 92.53)
20 min after pre-load	93.2 (88.13, 98.29)	88.3 (84.60, 91.92)
25 min after pre-load	93.2 (88.09, 98.38)	88.8 (84.69, 92.97)
30 min after pre-load	91.0 (86.80, 95.11)	88.3 (84.26, 92.35)

Note:

a Two-way ANOVA

**Table 6:** Comparison of HR between Volulyte 6% and Gelaspan 4% group with regards to time

Time	Mean (95% CI)^a^	
	Volulyte 6% Mean (95% CI) (*n* = 47)	Gelaspan 4% Mean (95% CI) (*n* = 46)
Baseline	81.9 (77.54, 86.20)	83.7 (80.23, 87.21)
5 min after pre-load	82.23 (77.22, 87.25)	83.2 (79.01, 87.21)
10 min after pre-load	81.6 (76.67, 86.48)	81.4 (77.27, 85.47)
15 min after pre-load	79.7 (74.84, 84.65)	78.6 (74.81, 82.41)
20 min after pre-load	78.7 (73.62, 83.70)	76.9 (72.74, 81.04)
25 min after pre-load	76.9 (71.83, 81.87)	75.5 (71.53, 79.52)
30 min after pre-load	77.8 (72.42, 83.24)	75.0 (70.79, 79.29)

Note:

a Two-way ANOVA

**Table 7:** Changes of acid-base balance and electrolytes in Volulyte 6% and Gelaspan 4% groups

Variable	Mean (SD) (*n* = 93)		*P*-value*
	Pre-treatment	Post-treatment	
pH			
Volulyte 6%	7.38 (0.04)	7.38 (0.04)	0.784
Gelaspan 4%	7.39 (0.03)	7.37 (0.04)	0.029^a^
HCO_3−_ (mmol/L)			
Volulyte 6%	24.35 (2.97)	25.00 (2.31)	0.233
Gelaspan 4%	24.27 (3.08)	25.61 (4.40)	0.050
BE (mmol/L)			
Volulyte 6%	1.13 (3.78)	1.49 (3.29)	0.618
Gelaspan 4%	0.87 (4.17)	2.44 (5.05)	0.062
Lactate (mmol/L)			
Volulyte 6%	1.21 (0.53)	1.19 (0.44)	0.774
Gelaspan 4%	1.24 (0.54)	1.86 (2.30)	0.061
Sodium (mmol/L)			
Volulyte 6%	135.53 (4.13)	134.85 (4.27)	0.222
Gelaspan 4%	136.41 (5.05)	136.91 (5.11)	0.556
Calcium (mmol/L)			
Volulyte 6%	0.88 (0.24)	0.97 (0.23)	0.070
Gelaspan 4%	0.98 (0.26)	1.09 (0.21)	0.017^a^
Potassium (mmol/L)			
Volulyte 6%	3.70 (0.41)	3.75 (0.64)	0.562
Gelaspan 4%	3.52 (0.55)	3.78 (0.38)	0.015^a^

Note: BE = base excess;

* Paired *t*-test,

a *P-*value < 0.05

**Table 8:** Comparison of acid-base and electrolyte changes between Gelaspan 4% and Volulyte 6%

Variable	Mean (SD)		*P*-value*
	Volulyte 6% (*n* = 47)	Gelaspan 4% (*n* = 46)	
pH			
Pre-	7.38 (0.04)	7.39 (0.03)	0.518
Post-	7.38 (0.04)	7.37 (0.04)	0.369
HCO_3−_ (mmol/L)			
Pre-	24.35 (2.97)	24.27 (3.08)	0.894
Post-	25.00 (2.31)	25.61 (4.40)	0.396
BE (mmol/L)			
Pre-	1.13 (3.78)	0.87 (4.17)	0.753
Post-	1.49 (3.29)	2.44 (5.05)	0.281
Lactate (mmol/L)			
Pre-	1.21 (0.53)	1.24 (0.54)	0.755
Post-	1.19 (0.44)	1.86 (2.30)	0.051
Sodium (mmol/L)			
Pre-	135.53 (4.13)	136.41 (5.05)	0.359
Post-	134.85 (4.27)	136.91 (5.11)	0.037^a^
Calcium (mmol/L)			
Pre-	0.88 (0.24)	0.98 (0.26)	0.062
Post-	0.97 (0.23)	1.09 (0.21)	0.018^a^
Potassium (mmol/L)			
Pre-	3.70 (0.41)	3.52 (0.55)	0.073
Post-	3.75 (0.64)	3.78 (0.38)	0.760

Note:

* Independent *t*-test;

a *P*-value < 0.05
